# HIV-1 infected humanized DRAGA mice develop HIV-specific antibodies despite lack of canonical germinal centers in secondary lymphoid tissues

**DOI:** 10.3389/fimmu.2022.1047277

**Published:** 2022-11-25

**Authors:** Matthew T. Ollerton, Joy M. Folkvord, Kristina K. Peachman, Soumya Shashikumar, Elaine B. Morrison, Linda L. Jagodzinski, Sheila A. Peel, Mohammad Khreiss, Richard T. D’Aquila, Sofia Casares, Mangala Rao, Elizabeth Connick

**Affiliations:** ^1^ Department of Medicine, University of Arizona, Tucson, AZ, United States; ^2^ Laboratory of Adjuvant and Antigen Research, United States Military HIV Research Program, Walter Reed Army Institute of Research, Silver Spring, MD, United States; ^3^ US Military Malaria Vaccine Program, Naval Medical Research Center, Silver Spring, MD, United States; ^4^ Diagnostics and Countermeasure Branch, Walter Reed Army Institute of Research, Silver Spring, MD, United States; ^5^ Department of Surgery, University of Arizona, Tucson, AZ, United States; ^6^ Department of Medicine, Feinberg School of Medicine, Northwestern University, Chicago, IL, United States

**Keywords:** humanized DRAGA mice, HIV - human immunodeficiency virus, secondary lymphoid tissue, germinal center (GC), follicle

## Abstract

A major barrier in the use of humanized mice as models of HIV-1 (HIV) infection is the inadequate generation of virus-specific antibody responses. Humanized DRAGA (hDRAGA) mice generate antigen-specific class switched antibodies to several pathogens, but whether they do so in HIV infection and the extent to which their secondary lymphoid tissues (sLT) support germinal center responses is unknown. hDRAGA mice were evaluated for their ability to support HIV replication, generate virus-specific antibody responses, develop splenocyte subsets, and organize sLT architecture. hDRAGA mice supported persistent HIV replication and developed modest levels of gp41-specific human IgM and IgG. Spleens from uninfected and HIV infected hDRAGA mice contained differentiated B and CD4^+^ T cell subsets including germinal center (GC) B cells and T follicular helper cells (TFH); relative expansions of TFH and CD8^+^ T cells, but not GC B cells, occurred in HIV-infected hDRAGA mice compared to uninfected animals. Immunofluorescent staining of spleen and mesenteric lymph node sections demonstrated atypical morphology. Most CD4^+^ and CD8^+^ T cells resided within CD20^hi^ areas. CD20^hi^ areas lacked canonical germinal centers, as defined by staining for IgD^-^Ki67^+^cells. No human follicular dendritic cells (FDC) were detected. Mouse FDC were distributed broadly throughout both CD20^hi^ and CD20^lo^ regions of sLT. HIV RNA particles were detected by *in situ* hybridization within CD20^+^ areas and some co-localized with mouse FDC. Viral RNA^+^ cells were more concentrated within CD20^hi^ compared to CD20^lo^ areas of sLT, but differences were diminished in spleen and eliminated in mesenteric lymph nodes when adjusted for CD4^+^ cell frequency. Thus, hDRAGA mice recapitulated multiple aspects of HIV pathogenesis including HIV replication, relative expansions in TFH and CD8^+^ T cells, and modest HIV-specific antibody production. Nevertheless, classical germinal center morphology in sLT was not observed, which may account for the inefficient expansion of GC B cells and generation of low titer human antibody responses to HIV-1 in this model.

## Introduction

A major hurdle for the use and interpretation of animal models in HIV-1 (HIV) pathogenesis research is the ability of these models to recapitulate key aspects of the infection. While simian immunodeficiency virus (SIV)- and simian human immunodeficiency virus (SHIV)-infected non-human primates (NHP) are the best models to date, NHP are costly, the viruses differ from HIV-1, and NHP have an immune system that differs from that in humans (1, 2). To address these problems, several humanized mouse models have been utilized to investigate HIV pathogenesis and assess antiretroviral therapies (3). While humanized mice harbor human immune cells and can be infected by HIV, a common limitation of most humanized mouse models is their inability to generate antigen-specific antibody responses (4).

The majority of antibody-producing cells mature within germinal centers (GC) of secondary lymphoid tissues (sLT), e.g., spleen, lymph nodes, and gut-associated lymphoid tissues (GALT) (5). Within GC, follicular dendritic cells (FDC) trap antigens, such as infectious HIV particles, on their cell surfaces in the form of complement opsonized immune complexes (6–8) and present them to B cells. Antigen-specific GC B cells engulf native antigen from FDC, and present antigen peptides to antigen-specific TFH that in turn produce cytokines such as interleukin (IL)-4 and IL-21 that promote B cell maturation (9, 10). These interactions result in class switching and affinity maturation of B cells and ultimately the generation of antibody producing plasma cells and antigen-specific memory B cells (11–14). Mice lacking functional GC fail to develop robust antibody responses (15–17). As TFH within GC and B cell follicles are major sites of HIV replication and FDC harbor large amounts of HIV-bound immune complexes *in vivo* (8), the lack of GC in humanized mice is a major limitation to their use in HIV immunopathogenesis studies.

One of the few humanized mouse strains that generates class switched antigen-specific antibodies is the DRAGA (HLA-A2. HLA-DR4. RAG1 KO. IL-2Rγc KO. NOD) mouse (18). These mice develop a human immune system by engraftment of human hematopoietic stem cells from cord blood. This model has been shown to generate antigen-specific antibodies to various pathogens including influenza A (19), scrub typhus (20), and protozoa (21). One study of humanized (h) DRAG mice (HLA-DR4. RAG1 KO. IL-2Rγc KO. NOD) (22), a predecessor of hDRAGA mice that lacks HLA-A2 molecules, demonstrated that these animals had high frequencies of TFH in the gut and female reproductive tract that were able to produce cytokines upon stimulation and were highly permissive to HIV (23). Evidence of antibody production in other infections as well as identification of HIV-infected TFH in the gut and female reproductive tract of the hDRAG mouse suggested that hDRAGA mice might have functional GC and consequently the ability to develop antibodies to HIV (23, 24). Whether hDRAGA mice are capable of developing organized GC to generate humoral immune responses to HIV is unknown.

In the present study, HIV-infected hDRAGA mice demonstrated sustained HIV replication and generated modest levels of HIV-specific antibodies. Within the spleen, relative expansions of TFH and CD8^+^ T cells were observed in HIV-infected mice compared to uninfected mice, similar to what is observed in sLT in HIV infection (25–27). No expansions in GC B cells were seen in HIV-infected hDRAGA spleen, unlike what is found in sLT in HIV infection (28). Spleens and mesenteric lymph nodes from hDRAGA mice failed to demonstrate many canonical features of human sLT architecture including defined B cell and T cell zones and structured GC. Human FDC were not detected in hDRAGA mouse spleen or mesenteric lymph nodes. Mouse FDC were detected, but demonstrated atypical distributions throughout sLT. HIV particles were detected in lymphoid tissue and in some instances were associated with mouse FDC, similar to what is seen in HIV infection. Frequencies of HIV RNA^+^ cells were elevated in CD20^hi^ regions, which is also seen in HIV infection. Nevertheless, after adjusting for percentages of CD4^+^ T cells, preferential accumulation of HIV RNA^+^ cells was attenuated in spleen and ablated in mesenteric lymph node, which is opposite to what is found in HIV infection (29). Thus, sLT in HIV-infected hDRAGA mice partially, but incompletely recapitulate sLT immunopathogenesis in HIV infection.

## Results

### hDRAGA mice engraftment and HIV infection

Eighteen HIV-infected hDRAGA mice were evaluated in this study (**Table 1**; **Supplementary Table 1**). hDRAGA mice were challenged with HIV 16 to 28 weeks after infusion of cord blood cells. The animals developed infection 7-28 days following intravaginal or intrarectal challenge with HIV. Three animals were euthanized during acute infection (8 to 15 days following infection) and the remainder were euthanized during chronic infection, i.e., ≥ 122 days after infection. Five mice were infected for 24 days, then received antiretroviral therapy (ART) for 42 days. ART was discontinued for 56 days prior to euthanasia. This cohort had similar viral loads and splenic architecture as untreated mice at the time of euthanasia and were thus included in our analyses (**Table 1** and **Supplementary Figure 1**). Nineteen uninfected hDRAGA mice were evaluated in this study. They were euthanized at a median of 258 days (range, 165 to 314 days) after infusion of cord blood cells, which was not significantly different from that of the chronically HIV-infected hDRAGA mice (median, 271 days; range, 199 to 284 days). All HIV-infected mice were female, as were 52% of HIV-uninfected mice.

**Table 1:** Animal characteristics.

Uninfected hDRAGA Mice				HIV Infected hDRAGA Mice				
Mouse #	Sex	Days post cord blood infusion		Mouse #	Sex	Days post cord blood infusion	Days post HIV infection	Plasma viral load at euthanasia log_10_
Z32	M	165		HD24 #4^3^	F	284	133	5.14
Z51	M	165		HD24 #5^3^	F	284	133	4.77
Z56	M	165		HD24 #6^3^	F	284	133	4.56
Z64	M	165		HD24 #13^3^	F	284	133	4.59
Z81	M	165		HD24 #15	F	199	8	0
Z960	M	257		HD24 #25	F	200	8	0
Z943	F	257		HD24 #26	F	215	15	4.84
Z169	F	125		47^2,4^	F	271	122	5.01
Z170	F	125		48^2,4^	F	271	122	4.66
Z805	F	314		50	F	236	122	4.71
Z925	F	314		53^2,4^	F	271	122	4.91
Z806	F	314		54^4^	F	236	122	5.46
Z807	F	314		59	F	271	122	3.91
Z752	F	258		51^1,2^	F	236	122	4.23
Z739^4^	M	299		52^1,4^	F	271	122	4.99
Z740^4^	M	299		55^1,2^	F	236	122	4.9
Z758^4^	F	299		57^1,2^	F	271	122	5.82
Z759^4^	F	299		58^1,2^	F	271	122	4.99
Z742^4^	M	299					

^1^Animals treated on ART for 42 days and off ART 56 days.

^2^Animals with mesenteric LNs.

^3^Animals with plasma antibody measurements over time.

^4^Animals with cryopreserved cells used for phenotypic analyses.

### HIV-infected hDRAGA mice develop sustained viremia and HIV-specific antibodies

**Figure 1:**
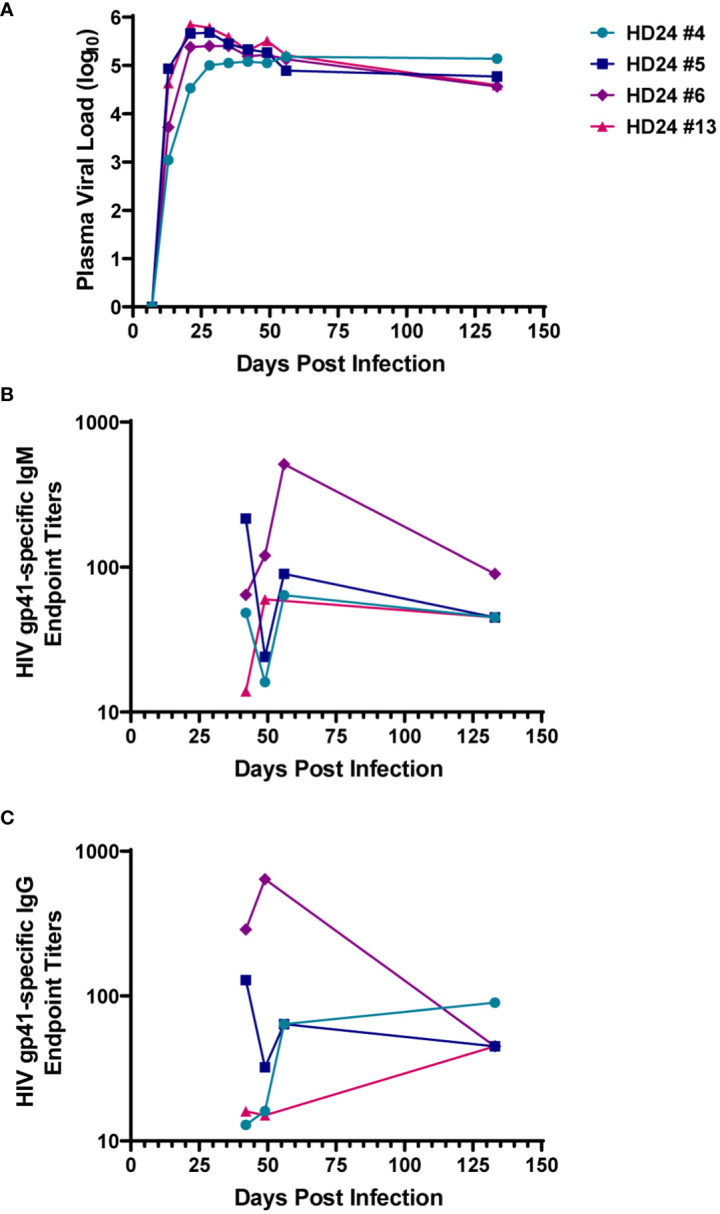
Humanized DRAGA (hDRAGA) mice sustain HIV replication and develop modest HIV-specific antibody responses. Four hDRAGA mice were infected with HIV and **(A)** plasma viral loads, **(B)** gp41-specific human IgM, and **(C)** gp41-specific human IgG end point titers were measured over time. Titers reported are mean values of duplicate wells unless insufficient volume was available then single data points were included.

Plasma HIV RNA and HIV-specific antibody responses were measured in four HIV-infected hDRAGA mice up to 133 days after infection (**Figure 1** and **Table 1**). All mice had detectable plasma viral loads beginning 13 days post infection (**Figure 1A**). Three of the four animals demonstrated peak viremia between days 21 and 28 after infection, then a subsequent 0.84 to 1.19 log_10_ decline in viral load at the time of euthanasia on day 133 post-infection. One mouse (HD24 #4) exhibited no decline in viremia and had the highest viral load at the time of euthanasia. The median viral load was 4.68 log_10_ (range 4.56-5.14 log_10_) at the time of euthanasia. Modest levels of HIV gp41-specific human IgM and IgG were detected at each time point measured between days 42 and 133 post-infection (**Figures 1B, C**, respectively).

### HIV infection alters human T cell compartments in spleens of hDRAGA mice

**Figure 2:**
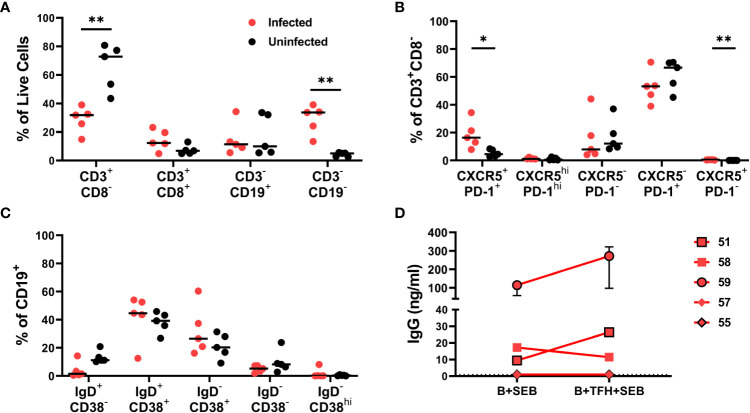
Phenotypic characteristics of lymphocyte populations in HIV infected and uninfected hDRAGA mice spleen. Cryopreserved spleen cells from five HIV uninfected and five infected hDRAGA mice were thawed and cultured overnight in R-15 media, stained for lineage markers, and analyzed by flow cytometry. **(A)** Percentages of lymphocyte and non-lymphocyte populations. **(B)** Percentages of CD3^+^CD8^-^ cell subsets defined by CXCR5 and PD-1 staining. **(C)** Percentages of CD19^+^ cell subsets that were naïve (IgD^+^CD38^-^), pre-GC (IgD^+^CD38^+^), germinal center (GC) B cells (IgD^-^CD38^+^), memory (CD38^-^IgD^-^), and plasmablast (IgD^-^CD38^hi^). **(D)** GC B and TFH cells were sorted from five cryopreserved HIV infected hDRAGA spleen cells and GC B were cultured with or without TFH (1:1) in R-10 in the presence of 100ng/ml SEB for 7 days. Cell culture supernatants were collected, and human IgG concentrations were quantified by ELISA. Statistical analyses were performed using Mann-Whitney tests (*p<0.05; **p ≤ 0.01).

Cryopreserved disaggregated spleen cells obtained at the time of euthanasia from an additional five HIV-infected and five uninfected hDRAGA mice were assessed for GC B and T cell phenotypes by flow cytometry (**Supplementary Figure 2** and **Table 1**). Because HIV downregulates CD4 on productively infected cells (30), CD4^+^ T cells were defined as CD3^+^CD8^-^ cells. Percentages of CD3^+^CD8^-^ cells were reduced in infected compared to uninfected mice (p=0.0079) (**Figure 2A**). CD3^+^CD8^-^ cells were further evaluated for the follicular markers CXCR5 and PD-1 (**Figure 2B**). Median percentages of TFH (CXCR5^+^PD-1^+^) cells were elevated in HIV-infected compared to uninfected mice (p=0.0156) while no statistically significant differences were detected for germinal center TFH (CXCR5^hi^PD-1^hi^) (**Figure 2B**). CXCR5^-^PD-1^+^ cells made up the majority of all splenic CD4^+^ T cells.

Phenotypic analyses of splenic B cells (**Figure 2C**) revealed that the IgD^+^CD38^+^ population including pre-GC B cells and the IgD^-^CD38^+^ population including GC B cells constituted the largest populations in both HIV-infected and uninfected hDRAGA mice. Few IgD^+^CD38^-^ cells, including naïve, IgD^-^CD38^-^ B cells including memory, and IgD^-^CD38^hi^ plasmablast B cells were detected in either HIV infected or uninfected hDRAGA mice. IgD^+^CD38^-^ B cells tended to be relatively reduced in infected compared to uninfected mice, although these differences were not statistically significant.

### GC B cells from HIV-infected hDRAGA mice produce IgG, which was augmented by TFH in some instances

To assess whether TFH could enhance GC B cell antibody production, previously cryopreserved splenocytes from five additional HIV infected animals were sorted for IgD^-^CD38^+^ GC B cells and TFH on a cell sorter. GC B cells were incubated with SEB in the absence or presence of TFH for seven days, and antibody production was assessed. Human IgG was detected in 3 of the 5 cultures (median range 9.4-272 ng/ml) (**Figure 2D**). In 2 of the 3 cultures with detectable antibody, the addition of TFH resulted in a 2.4 and 2.8 median fold increase in antibody concentrations. Thus, GC B cells from some hDRAGA mice were able to generate antibodies, and TFH in some instances could facilitate antibody production.

### hDRAGA mice lack conventional T and B cell compartmentalization in secondary lymphoid tissues

**Figure 3:**
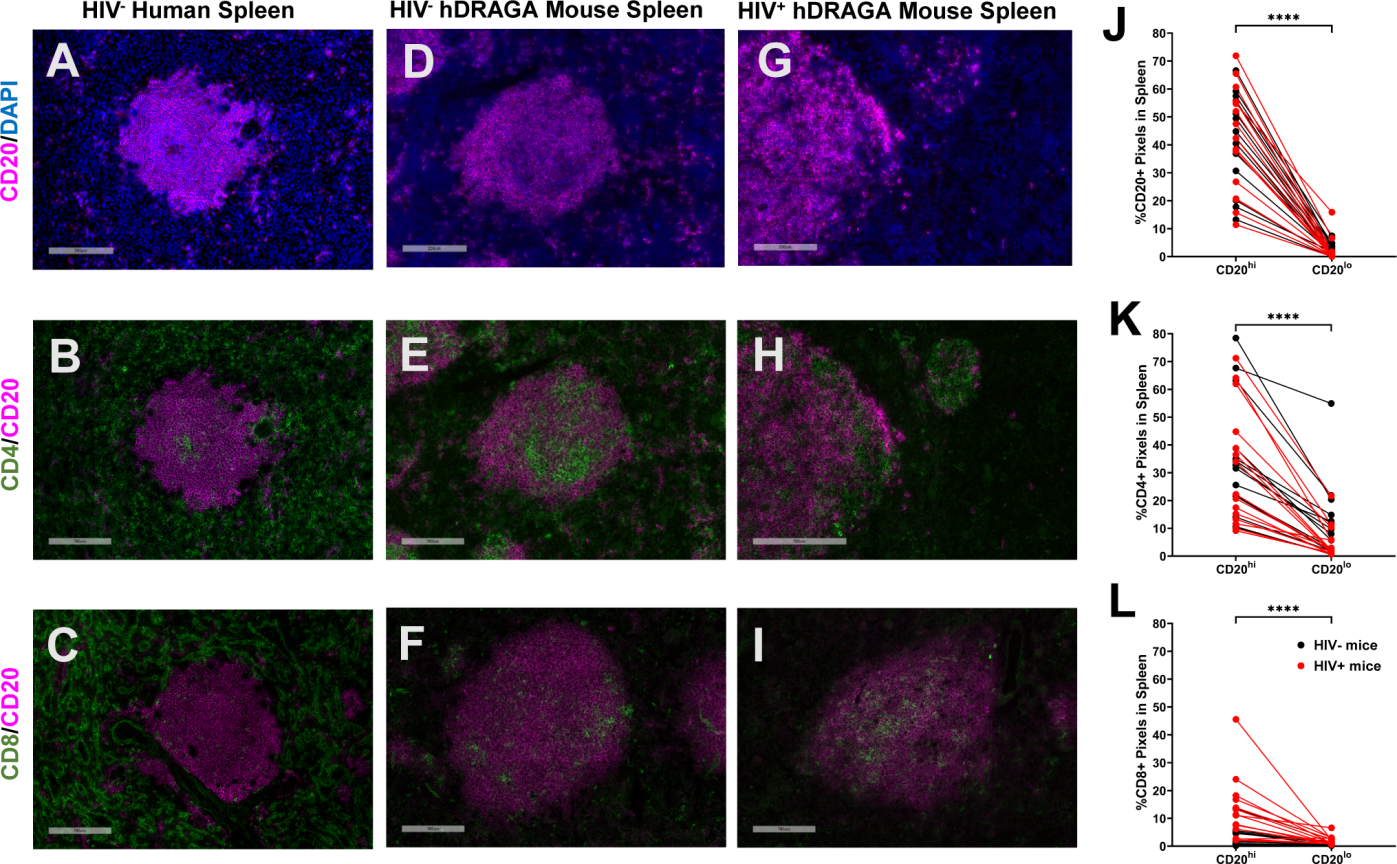
hDRAGA mice demonstrate abnormal lymphocyte organization in the spleen. Representative immunofluorescence images of **(A-C)** seven uninfected human spleen, **(D-F)** 14 uninfected, and **(G-I)** 18 HIV infected hDRAGA mice spleen stained for **(A, D, G)** CD20 (magenta) and DAPI (blue), **(B, E, H)** CD4 (green) and CD20 (magenta), and **(C, F, I)** CD8 (green) and CD20 (magenta). Bars equal 200µm. Percentages of **(J)** CD20+, **(K)** CD4+, and **(L)** CD8+ pixel area in CD20^hi^ and CD20^lo^ areas in 18 infected (red) and 14 uninfected (black) hDRAGA mice spleen. Statistical analyses were performed using Wilcoxon tests (****p ≤ 0.0001).

Spleen sections from six uninfected humans, 14 uninfected hDRAGA mice and 18 HIV-infected hDRAGA mice were evaluated by immunostaining for human CD20, CD4, and CD8 and representative images are shown in **Figures 3A-I**. In human spleen, CD20^hi^ areas, representing conventional B cell follicles (**Figure 3A**), were surrounded by the CD4^+^ and CD8^+^ rich T cell zone (**Figures 3B, C**, respectively) with occasional CD4^+^ cells and even rarer CD8^+^ cells observed inside the B cell follicle. In both HIV uninfected and infected hDRAGA mice, CD20^hi^ areas were also observed (**Figures 3D, G**, respectively). In contrast to human spleen, however, the majority of CD4^+^ (**Figures 3E, H**) and CD8^+^ cells (**Figures 3F, I**) were also found in the CD20^hi^ area of both uninfected and HIV-infected hDRAGA mice. Not only was CD20 staining elevated in the CD20^hi^ areas (**Figure 3J**), but staining for CD4 and CD8 was also significantly higher in the CD20^hi^ areas compared to CD20^lo^ areas in both uninfected and HIV-infected mice (**Figures 3K, L**, respectively). Analysis of CD3 co-expression in a subset of six uninfected and six HIV infected hDRAGA mice revealed that the vast majority of both CD4^+^ (median, 98%) and CD8^+^ cells (median, 100%) in spleen co-expressed CD3, indicating that they were T cells. Similar lack of conventional compartmentalization of B and T cells was also detected in mesenteric lymph nodes of seven HIV-infected hDRAGA mice (**Supplementary Figure 3**).

Because time since infusion of stem cells could potentially confound analyses, and there were no HIV uninfected mice at earlier time points, comparisons were made between splenic lymphocyte populations in a subset of 11 HIV-infected and seven uninfected hDRAGA mice, all of which had received stem cell infusions > 250 days prior (**Table 1**). Compared to HIV uninfected DRAGA mice, percentages of CD4^+^ area were reduced (p=0.0268) and percentages of CD8^+^ area were increased (p=0.0019) at euthanasia in the HIV^+^ DRAGA mice (**Supplementary Figure 4A, B**). Percentages of CD20^+^ area, on the other hand, remained low (median, 6%) and were not significantly different between uninfected and infected mice (**Supplementary Figure 4C**).

### hDRAGA mice lack canonical GC and FDC networks

**Figure 4:**
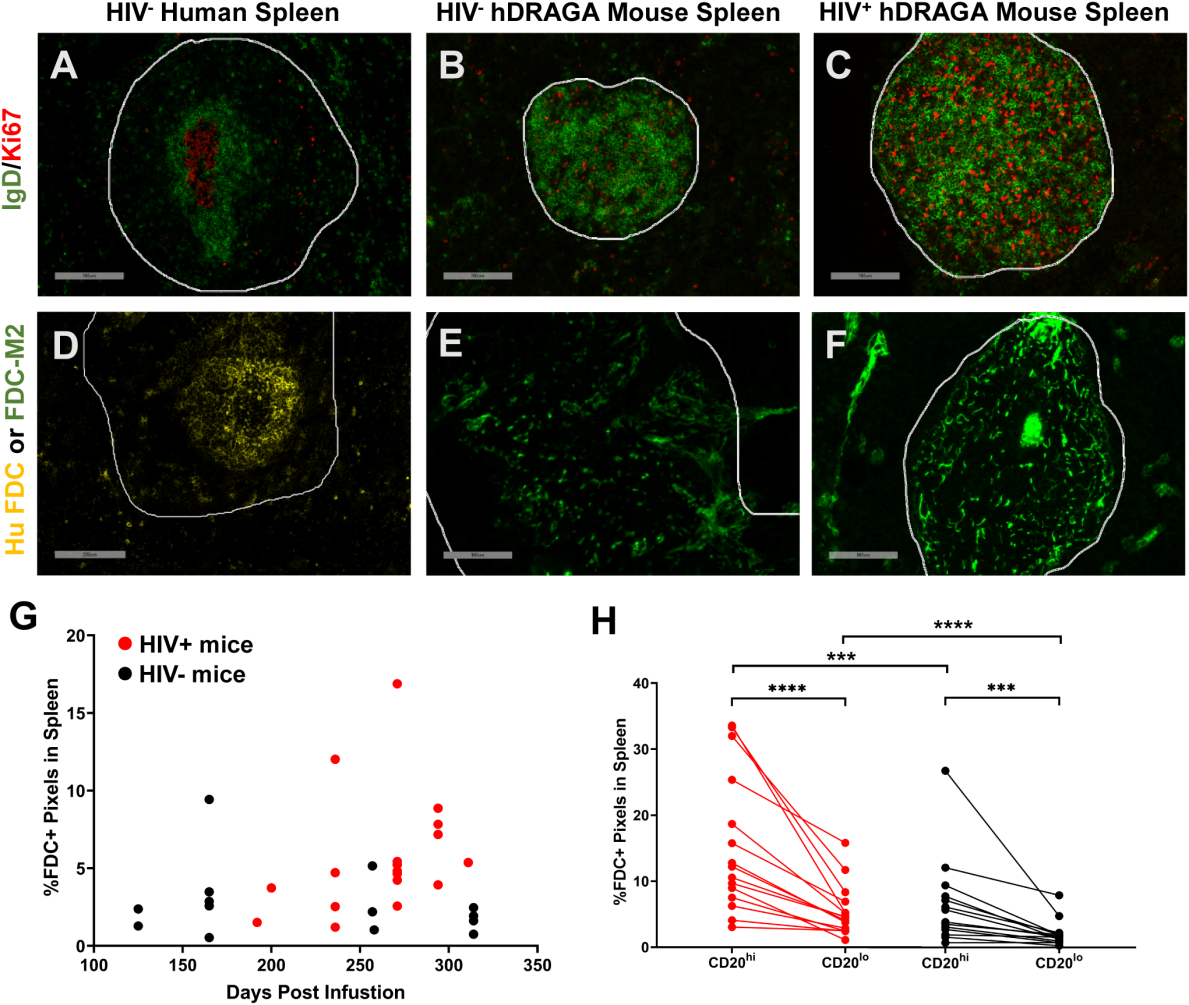
hDRAGA mice spleen do not form traditional germinal centers and lack canonical FDC distribution. **(A-C)** Representative immunofluorescence images of Ki67 (red) and IgD (green) staining in **(A)** seven uninfected human spleen, **(B)** 14 uninfected, and **(C)** 18 HIV infected hDRAGA mouse spleen. Bars equal 200 µm. **(D-F)** Representative immunofluorescence images of FDC staining in **(D)** seven uninfected human spleen using human FDC antibody (yellow), and **(E)** 14 uninfected and **(F)** 18 HIV infected hDRAGA mouse spleen using mouse FDC antibody (green). Bars equal **(D)** 200 µm and **(E, F)** 100 µm. CD20 staining is not shown. CD20^hi^ areas are demarcated by white lines. **(G)** Total percentage of mouse FDC+ pixel area, as determined by quantitative image analysis, in HIV infected (red; n=18) and uninfected (black; n=14) hDRAGA mice spleen was plotted against time post infusion. **(H)** Percentages of mouse FDC+ pixel area in CD20^hi^ and CD20^lo^ areas in HIV infected (red; n=18) and uninfected (black; n=14) hDRAGA mice spleen. Statistical analyses were performed using a Wilcoxon test (***p≤0.001; ****p≤0.0001) and Mann-Whitney tests (***p≤0.001; ****p≤0.0001).

GC within secondary B cell follicles are readily identified histologically by the presence of IgD^-^Ki67^+^ B cells surrounded by a ring of IgD^+^ B cells, as shown in a representative example of a spleen from a human without HIV infection (**Figure 4A**). Both Ki67^+^ and IgD^+^ cells were detected in spleen of 14 uninfected and 18 HIV infected hDRAGA mice and were located primarily within the CD20^hi^ areas. Nevertheless, no structured GC were observed in either HIV-uninfected or infected mice as shown in representative images (**Figures 4B, C**, respectively). Instead, Ki67^+^ cells and IgD^+^ cells were distributed throughout the CD20^hi^ areas. Conventional GC morphology, as shown in a representative image from an inguinal lymph node of a person living with HIV (**Supplementary Figure 5A**), was also absent in mesenteric lymph nodes from seven HIV-infected hDRAGA mice, which demonstrated a generalized nonfocal distribution of Ki67^+^ cells and IgD^+^ cells in CD20^hi^ areas, as shown in a representative image (**Supplementary Figure 5B**).

Human and mouse FDC are located almost exclusively within GC and appear histologically as focal, lattice like structures within B cell follicles, as shown in representative images of a spleen from a human without HIV infection (**Figure 4D**) and a spleen from a C57/BL6 mouse (**Supplementary Figure 6A**). No human FDC were detected in any of the 14 uninfected and 18 HIV-infected hDRAGA mice by immunostaining (representative image of negative staining, **Supplementary Figure 6B**); mouse FDC were identified in spleens from all HIV uninfected and HIV infected hDRAGA mice, but failed to demonstrate conventional focal networks as shown in representative images (**Figures 4E, F**, respectively). Instead, all FDC were of mouse origin and found to be dispersed throughout or concentrated at the periphery of the CD20^hi^ area (additional images are shown in **Supplementary Figures 6C-G**). Total splenic mouse FDC pixel area tended to increase over time in infected animals, but not in uninfected mice (**Figure 4G**). Although mouse FDC were found primarily within CD20^hi^ areas (p<0.0001), a substantial percentage of the FDC were detected unexpectedly outside of CD20^hi^ areas of the spleen (**Figure 4H** and **Supplementary Figures 6C-G**). Compared to uninfected mice, HIV infected mice had a greater percentage of FDC staining area in both CD20^hi^ areas (p=0.0027) and in CD20^lo^ areas of spleen (p=0.0005). Aberrant distribution of FDC was also observed in mesenteric lymph nodes of hDRAGA mice. Unlike the typical organization of FDC in lymph nodes from people living with HIV, as shown in a representative image (**Supplementary Figure 5C**), mouse FDC were dispersed throughout mesenteric lymph nodes of seven HIV-infected hDRAGA mice, as shown in a representative image (**Supplementary Figure 5D**).

### HIV RNA^+^ cells and HIV RNA particles are detected within lymphoid tissue of hDRAGA mice

**Figure 5:**
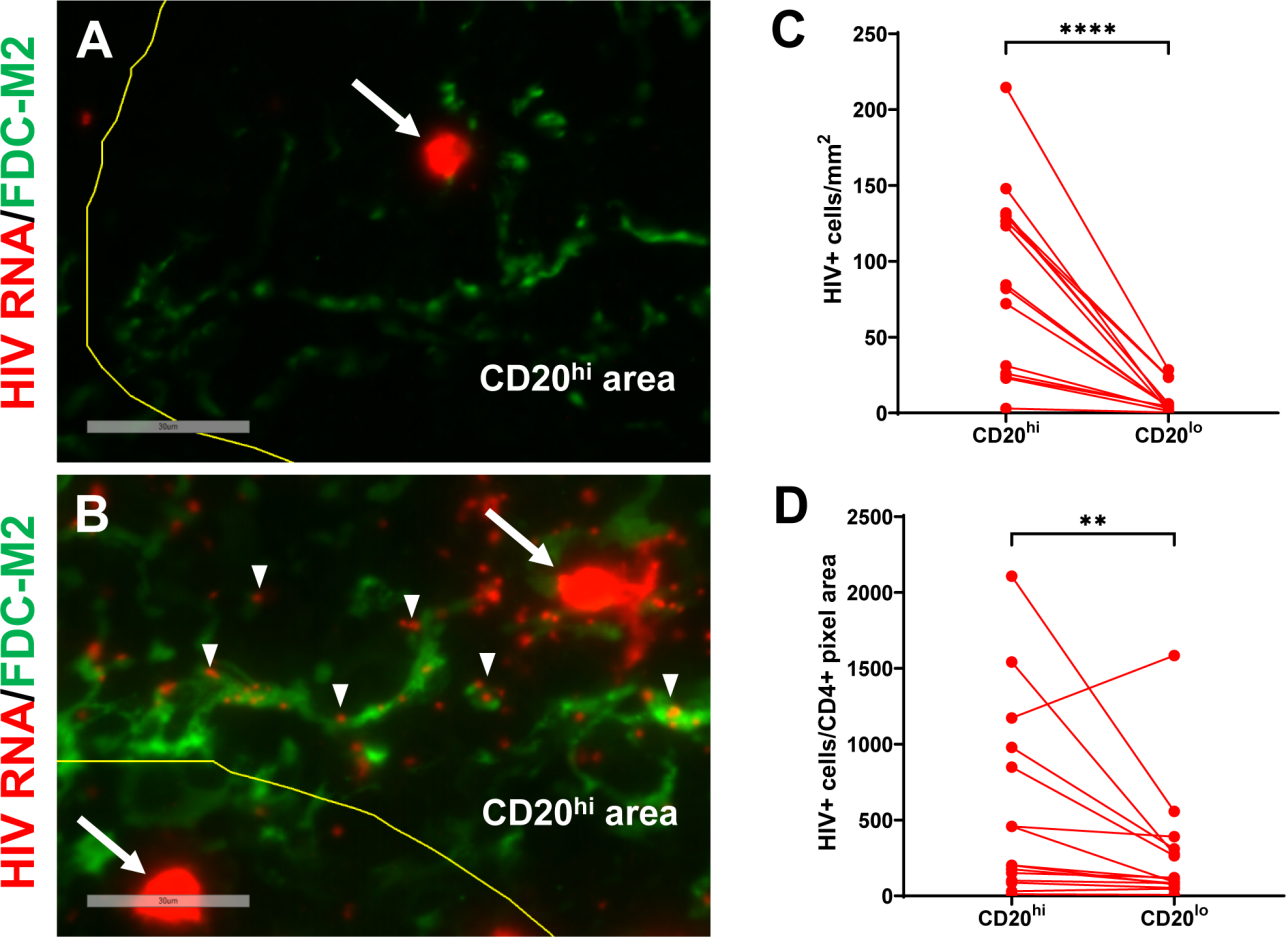
HIV vRNA+ cells and vRNA particles are detected in spleen of infected hDRAGA mice. Representative images of *in situ* hybridization for HIV RNA (red) in spleen sections from **(A)** three acutely infected (shown, HD24 #26), and **(B)** 15 chronically infected (shown, HD24 #4) hDRAGA mice. Mouse FDC are shown in green. CD20 staining is not shown. CD20^hi^ areas are demarcated by yellow line. vRNA^+^ cells are marked by arrows and representative individual HIV particles are indicated by arrowheads. Bars equal 30 µm. **(C)** Frequencies of vRNA^+^ cells were quantified in CD20^hi^ areas and CD20^lo^ by visual inspection and quantitative image analysis and **(D)** adjusted for CD4^+^ pixel area (n=15). Statistical analyses were performed using a Wilcoxon test (****p ≤ 0.0001; **p ≤ 0.01).

Spleens from 3 acutely HIV infected (8 to 15 days post-infection), 15 chronically HIV infected (all ≥ 120 days post infection), two uninfected hDRAGA mice, and mesenteric lymph nodes from seven HIV-infected hDRAGA mice were analyzed for the presence of HIV RNA by *in situ* hybridization. HIV RNA^+^ (vRNA^+^) cells defined by circular areas of cell-associated high intensity staining were readily detected in spleen and mesenteric lymph nodes of HIV infected DRAGA mice, as indicated by arrows in representative images (**Figures 5A, B** and **Supplementary Figure 7A**), but not detected in uninfected mice. Frequencies of vRNA^+^ cells/mm^2^ were elevated within CD20^hi^ areas compared to CD20^lo^ areas in spleen (**Figure 5C**) and mesenteric lymph nodes (**Supplementary Figure 7B**). After adjusting for the frequency of CD4^+^ cells within the two compartments, however, these differences were less marked in spleen (p=0.0067, **Figure 5D**) and no differences were detected in mesenteric lymph nodes (**Supplementary Figure 7C**).

Spleens and mesenteric lymph nodes were also evaluated for the presence of viral RNA particles, which are readily distinguished from vRNA^+^ cells by their small size. No viral RNA particles were detected in uninfected hDRAGA mice. In three acutely infected hDRAGA mice (8-15 days post infection), HIV RNA particles were rarely detected in the spleen and none associated with FDC (**Figure 5A**), despite the fact that vRNA^+^ cells were abundantly present. In spleens and mesenteric lymph nodes from chronically HIV infected hDRAGA mice, HIV RNA particles were frequently observed and some localized with FDC, as shown in representative images by arrowheads (**Figure 5B** and **Supplementary Figure 7A**).

## Discussion

This is the first study to evaluate HIV replication, HIV-specific antibody production, and sLT organization in hDRAGA mice. Declines in viremia following acute HIV infection and relative expansion of TFH and CD8^+^ T cells in these mice parallel clinical aspects of the infection seen in humans (25, 31, 32). Modest levels of HIV-specific antibody were generated in HIV-infected hDRAGA mice, and TFH from spleens of some of the infected hDRAGA mice promoted IgG production ex vivo. Nevertheless, spleens and mesenteric lymph nodes from these animals lacked distinct B and T cell zones and canonical GC organization with distinct segregation of IgD^+^ and Ki67^+^ cells within B cell follicles. Only FDC of mouse origin were detected and they demonstrated aberrant distribution both within and outside of CD20^hi^ areas. HIV RNA particles were detected in hDRAGA mice spleens and mesenteric lymph nodes and some particles localized with mouse FDC during chronic, but not acute infection, similar to what is reported in SIV-infected rhesus macaques (33–35). HIV RNA^+^ cells were more concentrated in CD20^hi^ areas than CD20^lo^ areas, but after adjusting for numbers of CD4^+^ cells these differences were attenuated in spleen and ablated in mesenteric lymph node, unlike what has previously been reported in HIV and SIV infection (29, 36). Thus, HIV-infected hDRAGA mice recapitulate many, but not all aspects of human HIV infection and lack typical sLT organization.

Both HIV infected and uninfected hDRAGA mice had high percentages of T cells that expressed PD-1 in tissue, as has been reported in other models (23, 37–39). Given the xenograft nature of the model, human T cells may be chronically stimulated by mouse cells. Alternatively or in addition, homeostatic proliferation, which occurs in the context of lymphopenia and has been associated with a subset of PD-1-expressing cells, might also contribute (40).

The generation of antigen-specific class switched antibody sets the hDRAGA mouse apart from many other humanized mouse models (4). In the present study, HIV-infected hDRAGA mice developed modest levels of HIV-specific human IgM and IgG, similar to what was previously seen in hDRAG mice (22), but lower levels than are typically seen in people living with HIV. Limitations of this study are that relatively small sample sizes were used for antibody titers and flow cytometry analyses, and the neutralizing capacity of the HIV-specific antibody produced in hDRAGA mice was not assessed. Future studies within this model to evaluate HIV neutralizing antibody could be informative.

Multiple lines of evidence indicate that suboptimal antibody development was likely related to impairment of the GC reaction. Relative expansions of TFH and CD8^+^ T cells suggest that effective antigen stimulation of T cells occurred in hDRAGA mice. Nevertheless, there was not a significant expansion in GC TFH nor differentiated B cell populations in HIV infected hDRAGA compared to uninfected mice. Furthermore, there was no expansion of CD20^+^ areas in spleens of HIV infected compared to uninfected hDRAGA mice, in contrast to the marked follicular hyperplasia that is seen in chronic HIV and SIV infection (41, 42). Collectively, these data suggest impairment of the GC reaction.

Failure to form canonical GC is the most likely explanation for impaired GC reactions and reduced antigen specific antibody generation in the hDRAGA mice. Similar findings of disorganized lymphoid tissue architecture and lack of canonical GC have been observed in other humanized mice (4, 43–45). B cells, TFH, and FDC are all required for the formation of GC within B cell follicles; animals that lack any one of these cell types do not form normal GC (46). Lack of GC formation in hDRAGA mice, as well as other humanized mouse models, is most likely related to absence of human stromal cells such as FDC and their precursors (4). Although mouse FDC were present in hDRAGA mice, they may be unable to induce GC formation and may not provide the costimulatory signals needed to efficiently induce antigen-specific B cells and promote TFH helper functions. A recent study comparing human and mouse FDC transcriptomes noted conservation of many, but not all, expressed genes (47). FDC originate from mesenchymal stem cells; consequently, one would not expect human FDC to develop in humanized mice reconstituted with human hematopoietic stem cells. Interestingly, administration of bone marrow-derived mesenchymal stem cells to rhesus macaques during SIV infection resulted in restoration of B cell follicles and FDC within gut associated lymphoid tissue (48). Whether engraftment of human mesenchymal cells or expression of select human FDC proteins by humanized mice FDC results in GC formation and improved antibody responses in hDRAGA mice warrants further examination.

FDC are an important site of HIV accumulation in individuals with untreated chronic HIV infection (8, 49, 50). SIV and HIV RNA particles accumulate on FDC in large numbers following development of antibody, as they are attached to FDC *via* antibody binding to Fc receptors (33–35, 51). HIV RNA particles remain elevated on FDC until antiretroviral therapy is initiated, or GC involute in advanced disease (41, 52). Similar to HIV and SIV infection (34, 51), very little extracellular virus was detected in lymphoid tissues at 8 to 15 days post infection in hDRAGA mice. This was likely due to lack of abundant antibody responses at that time, although antibody levels were not measured in these specific animals. In chronic HIV infection of hDRAGA mice, FDC associated virions were found in many instances, indicating that mouse FDC could bind human antibody associated HIV immune complexes. Nevertheless, how efficiently mouse FDC bind and retain human antibody-antigen complexes is unclear.

HIV and SIV replication are concentrated in TFH in sLT in chronic untreated infections in humans and rhesus macaques, respectively, even though B cell follicles have fewer target cells than the adjacent T cell zone (36, 53). After adjusting for target CD4^+^ T cell density in B cell follicles and extrafollicular areas, preferential infection of CD4^+^ cells in B cell follicle is even more pronounced in humans living with HIV and SIV-infected rhesus macaques (29, 36). Preferential infection of TFH in B cell follicles likely is related to high permissivity of TFH, the presence of infectious virions on FDC, and the relative paucity of virus-specific CD8^+^ T cells in B cell follicles compared to extrafollicular areas (54). In HIV infected hDRAGA mice, HIV RNA^+^ cell numbers were elevated in CD20^hi^ areas; however, when CD4^+^ T cell density was considered, the tendency for preferential infection within CD20^hi^ areas was reduced in spleen and ablated in mesenteric lymph nodes. This could be related to lack of the typical distribution of FDC and TFH. Intriguingly, CD8^+^ T cells were also highly concentrated within CD20^hi^ areas. This finding is somewhat counter to the argument that we have previously made that restriction of CD8^+^ T cells from B cell follicles and GC is a major cause for persistent HIV replication, although the effector capability of these CD8^+^ T cells is unknown. Future studies to examine the magnitude and effectiveness of HIV-specific CD8^+^ T cell responses and the impact of CD8^+^ T cell depletion on virus expression in this model could provide additional insight into this issue.

Humanized mouse models provide an attractive alternative to nonhuman primate models due to their affordability and relative ease of manipulation. Evaluation of various antiretroviral strategies including latency reversal and passive antibody administration is feasible in hDRAGA mice as they contain human immune cells. Furthermore, these mice could be tested for vaccine efficacy of the passively administered antibodies as they express human FcRs which could internalize HIV-immune complexes for processing of HIV antigens and presentation of peptides to human T cells. The passively administered antibody could also trigger antibody-dependent cytotoxicity. HIV-infected hDRAGA mice recapitulate many clinical aspects of HIV-1 infection despite noncanonical lymphoid tissue organization. Modest virus-specific antibody responses were generated, which is a significant improvement over most other humanized mouse models of HIV infection. Nevertheless, lack of structured germinal centers and resulting inefficient GC reactions remain a limitation of this model for studying HIV infection as GC are central to HIV immunopathogenesis. Reconstitution of human FDC or human FDC functions in the hDRAGA mouse and other humanized mouse models could potentially lead to canonical GC formation, and enhanced utility in studies of HIV immunopathogenesis.

## Materials and methods

### Humanized DRAGA mice and ethics statement

DRAGA mice express the HLA-A2.1 and HLA-DR0401 transgenes on a Rag1KO.IL2RγcKO.NOD (NRG) background, and they have been described previously (18–21). De-identified umbilical cord bloods positive for HLA-A2.1 and HLA-DR0401 were commercially procured through the New York Blood Center (Long Island City, NY, USA (https://nybloodcenter.org/products-and-services/blood-products/research-products/). Mice were bred at the Veterinary Service Program at WRAIR/NMRC. Eight- to twelve-week-old mice were irradiated (350 rads) and injected intravenously with CD3^+^ T cell-depleted cord blood cells (EasySep Human CD3 Positive Selection Kit, cat#18051, Stem Cell Technologies) containing approximately 10^5^ human CD34^+^ hematopoietic stem cells (HSC) as determined by FACS using a mouse anti-human CD34 antibody (BD Biosciences, cat#550761). The procedures for assessing percentages of human T and B cells by FACS in peripheral blood have been previously described (18–21). As documented in our previous studies, >95% of HSC-infused DRAGA mice reconstitute a human immune system by 3 to 4 months post-CD34^+^ HSC infusion (18–21). All animal procedures reported herein were conducted under IACUC protocols approved by WRAIR/NMRC (#19-IDD-24 & #19-RET-31) in compliance with the Animal Welfare Act and in accordance with the principles set forth in the “Guide for the Care and Use of Laboratory Animals,” Institute of Laboratory Animals Resources, National Research Council, National Academy Press, 2011.

### HIV infection

HIV infection was carried out as previously described (23, 24). Briefly, DRAGA mice were injected subcutaneously with medroxyprogesterone (2.5 mg per 50 μl per mouse) (Greenstone LLC) 7 days prior to infection. Mice were anesthetized and purified primary HIV-1 BaL (10,000 TCID50, ~2.54 ng p24) in a total volume of 20 μl was administered intravaginally. HIV-1 BaL was purified and quantified as described previously (55). Blood samples (30 μl) were collected from hDRAG mice pre- and post-infection in tubes containing 20 μl 18 mM EDTA solution. Following centrifugation at 3,300 rpm for 10 min at 4°C, plasma and the cell pellet were separately stored frozen at −20°C.

HIV viral loads were determined using a modification of the COBAS^®^ AmpliPrep/COBAS^®^ TaqMan HIV-1 Test v2.0. Briefly, plasma samples are thawed at room temp (RT) just prior to testing. SPEX buffer was added to 30ul of thawed plasma sample, pulse vortexed to mix, transferred to a tube containing additional SPEX buffer and tested according to manufacturer instructions. HIV values were adjusted by multiplication with the dilution factor (35). The assay lower limit of detection using 30ul of plasma was 700 copies/mL.

### Antiretroviral therapy

A subset of hDRAGA mice were placed on a 3 drug ART regimen (tenofovir (5.35mg), emtricitabine (0.25mg), raltegravir (2.75mg/day target) 24 days after HIV infection (manuscript under preparation). ART was dissolved in Medidrop Sucralose (sweet water) mixed with acidified water. ART/water mix was replaced twice weekly for 6 weeks. At day 42, animals were taken off of ART. At day 122 (56 days after ART was stopped) blood, spleen, and mesenteric lymph nodes were collected.

### Detection of gp41-specific human IgM and IgG

gp41-specific antibodies were determined by ELISA as previously described with some modifications (56). Briefly, Immulon 2 U-bottom 96-well ELISA plates (Thermo Scientific) were coated overnight at 4°C with 0.1ug/mL of full-length subtype B gp41 protein diluted in PBS. The protein was produced, purified and kindly provided by V. B. Rao from The Catholic University of America (57). Plates were washed three times with washing buffer (PBS containing 0.1% Tween 20) and blocked overnight with blocking buffer (0.5% Milk in PBS with 0.1% Tween 20) at 4°C. Dilutions of the serum samples in blocking buffer were added to the wells of the plate and the plate was incubated at room temperature for one hour. Dilutions depended on serum availability but were typically performed in the range of 1:16-1:64. Plates were washed 3 times in washing buffer followed by the addition of secondary antibody, horseradish peroxidase labeled sheep anti-human IgM or anti-human IgG antibody (The Binding Site) at a dilution of 1:1,000 in blocking buffer for 1 hour at room temperature. After washing 3 times, ABTS substrate from KPL was added for 1 hour and the plates were read at 405nM on a SpectraMax plate reader. Human HIVIG was used as a positive control for the plates. OD for the buffer control for p24 ELISA was (IgM=0.07; IgG=0.105). The endpoint titer is calculated as equal to twice the buffer control. The IgM and IgG OD for infected animals ranged from 0.15-0.46 and 0.23-0.28 respectively. Similarly, the OD for the buffer control for gp41 ELISA was 0.08 for both IgM and IgG. The IgM and IgG OD for infected animals ranged from (0.25-1.60) and (0.17-0.69) respectively. Most endpoint titers titrated down several wells before reaching values equal to twice background. The end point titer is defined as the highest reciprocal serum dilution that yielded an absorbance twice the background values (wells without primary antibody).

### Cryopreservation of hDRAGA mouse spleen cells and tissue

Prior to tissue collection, approximately 1 mL of blood was collected by cardiac puncture. The following tissues were obtained from the hDRAGA mice: spleen and mesenteric lymph nodes. A part of the spleen and several lymph nodes were also flash frozen on dry ice ethanol in OCT freezing media for histochemical analysis before being stored at -80°C until analysis. The remaining spleen and lymph nodes were placed in separate tubes containing 1× HBSS (Ca++ and Mg++ free), 1×HEPES, 5% FBS (vol/vol) wash buffer on ice. Single cells from the spleen and lymph nodes were isolated as previously described (23). Briefly, single cells were generated by gently mashing the spleen using the rubber stopper end of a 1 mL syringe followed by passing through a 0.45µm strainer. Cells were washed and pellets were resuspended in 1 mL 90% FBS/10% DMSO and frozen at -80°C overnight before being placed in a liquid N_2_ freezer for long-term storage.

### Phenotyping of hDRAGA spleen cells by flow cytometry

Cryopreserved spleen cells from uninfected and HIV infected hDRAGA mice were thawed and cultured overnight in R-15 media (RPMI 1640 supplemented with 15% heat inactivated FBS (Sigma), 1X nonessential amino acids (Gibco), 1X Glutamax (Gibco), 1X Primocin (*In vivo*gen), and 100 units Benzonase (Sigma)). Cells were centrifuged at 300xg for 10 minutes, then resuspended in an antibody cocktail consisting of CD3-AF700 (clone SP34-2; Becton Dickinson (BD)), CD19-PeCy7 (Clone SJ25C1; Tonbo), IgD-FITC (Clone IA6-2; BioLegend), CD8-BV510 (clone RPA-T8; BD); CXCR5-PE (clone MU5UBEE; Invitrogen), PD-1-BV785 (clone EH12.2H7; BioLegend), CD38-APC (clone HIT2; Tonbo), and Brilliant Stain Buffer (BD). Cells were labeled for 20 minutes at room temperature in the dark, then washed with PBS without calcium and magnesium, then centrifuged at 300xg for 10 minutes. Pellets were resuspended in PBS supplemented with 2% FBS and 1mM EDTA and 7-AAD (Tonbo) was added. Cells were passed through a 40 μm filter cap and analyzed on an LSR Fortessa (BD) flow cytometer.

### Antibody production from GC B and TFH cell culture

GC B cells (7-AAD^-^CD19^+^CD38^+^IgD^-^) and TFH (7-AAD^-^CD3^+^CD8^-^CXCR5^+^PD-1) cells were sorted on a FACSAria III. Sorted TFH and GC B cells (2x10^4^ cells each) were added to the wells of a 96-round bottom plate as indicated. Cells were cultured in R-10 (RPMI-1640 supplemented with 10% heat inactivated FBS (Sigma), 1X nonessential amino acids (Gibco), 1X Glutamax (Gibco), 1X Primocin (*In vivo*gen), and 100 ng/ml Staphylococcal enterotoxin B (SEB; Listlabs) in a final volume of 100 μl/well and incubated at 37°C and 5% CO_2_ for seven days. Plates were centrifuged at 800xg for 10 minutes, then the supernatant was collected from each well and stored at -80°C. Human IgG was quantified from cell culture supernatants by ELISA (StemCell) according to the manufacturer’s instructions and measured on a plate reader at 405 nm using a correction wavelength of 650 nm. Human IgG concentrations were calculated from a standard curve generated from each plate using the human IgG standard provided.

### Human tissue

Archived inguinal lymph nodes obtained from people living with HIV-1 who were not receiving antiretroviral therapy were obtained as previously described (58, 59) and used with permission from the University of Arizona’s IRB. Normal human spleen tissue was obtained from patients undergoing splenectomies during distal pancreatectomy performed for benign and malignant diseases of the pancreas. Tissue was collected as soon after surgery as possible as a 1 cm block, embedded in OCT, then frozen by the University of Arizona Cancer Center’s Tissue Acquisition Core. The University of Arizona IRB determined that the proposed collection was non-human subject’s research as defined by DHHS and FDA regulations.

### Immunofluorescent staining of spleen and lymph node tissue sections

Slides of 6 μm thick sections of frozen tissue were fixed in 1% paraformaldehyde for 20 min, then stained for 1 hour with primary antibodies. Primary antibodies directed at human antigens included: Rabbit anti CD20 (Abcam), Rat anti CD4 (antibodies on-line, cloneYNB46.1.8), Mouse anti Ki-67 (BD, clone B56), Rabbit anti IgD (Abcam, clone mAb EPR6146), Rabbit anti CD8 (Abcam, clone mAb 144B) and Mouse anti FDC (Invitrogen, clone CNA.42). Primary antibodies directed at mouse antigens included: Rat anti FDC-M2 (Immuno Kontact, clone FDC-M2). After washing, sections were incubated with appropriate fluorescent secondary antibodies (Invitrogen) for 30 min. In some cases, CD20, directly labeled using a Zenon labelling Kit (Invitrogen), was added and incubated for an additional hour. All slides were counterstained with DAPI; whole sections were imaged using an Aperio Versa Slide Scanning System (Leica) at 40X.

### *In situ* hybridization for HIV RNA

Slides of 6 μm thick sections of frozen spleen and lymph node tissue were fixed in 4% paraformaldehyde for 30 min. Sections were treated for 10 min. with 2% hydrogen peroxide followed by *in situ* hybridization using Advanced Cell Diagnostics’ branched DNA technique (RNAscope). HIV RNA^+^ cells and particles were detected using TSA Plus Cy3 System (Perkin Elmer). Sections were incubated overnight at 4˚C with Rabbit anti CD20 (abcam) and Rat anti FDC-M2 (Immuno Kontact, clone FDC-M2). Primary antibodies were detected with appropriate fluorescent secondary antibodies (Invitrogen) for 1 hour. All slides were counterstained with DAPI and whole sections were imaged using an Aperio Versa Slide Scanning System (Leica) at 40X.

### Quantitative image analysis of spleen and lymph node sections

Using Aperio Imagescope (v12.4.0.5043, Leica) CD20^hi^ areas were defined by clusters of CD20 staining and percent area of mouse FDC, human CD4 and human CD8 were determined using a positive pixel count algorithm (Leica). HIV RNA^+^ cells were quantified for both CD20^hi^ and CD20^lo^ regions. Total and CD20^hi^ areas were determined using Aperio Imagescope and frequency of HIV RNA^+^ cells per square millimeter was calculated. Statistical analyses were conducted using Prism 9.3.1 (GraphPad).

## Data availability statement

The original contributions presented in the study are included in the article/**Supplementary Material**. Further inquiries can be directed to the corresponding author.

## Ethics statement

The animal study was reviewed and approved by Walter Reed Army Institute of Research/Naval Medical Research Center (#19-IDD-24 & #19-RET-31).

## Author contributions

RD, EC, SC, MR, MO, and JF were involved in the design of the experiments. Funding was provided from RD and SP. SC and SS bred and generated DRAGA mice. EM and KP performed the HIV infection and ART studies, antibody studies and collected animal tissues. LJ and SP provided viral load data. MR, KP, and SC analyzed, compiled, and interpreted infection, animal, and antibody data. MK provided human spleen tissues. JF performed all tissue staining and image analyses, MO performed all flow cytometry staining, and cell culture experiments. All authors contributed to the article and approved the submitted version.

## Funding

Collection of normal human spleen specimens and utilization of the University of Arizona Cancer Center Flow Cytometry Shared Resource as reported in this publication were supported by the National Cancer Institute of the National Institutes of Health under award number P30 CA023074. Support for MO was provided by the Moya-Teller Fund. This work was partially supported as a subaward on P01AI131346-04 grant awarded to RD at Northwestern University, Chicago.This work is supported in part by the US Army Medical Research and Development Command under Contract No. W81-XWH-18-C-0337 (formerly W81-XWH-16-C-0225) and by the US Military HIV Research Program, Walter Reed Army Institute of Research under a cooperative agreement (W81XWH-18-2-0040, formerly W81XWH-11-2-0174) between the Henry M. Jackson Foundation for the Advancement of Military Medicine, Inc. (HJF), and the US Department of Defense (DoD). The views, opinions and/or findings are those of the authors and should not be construed to represent the positions, policy or decision of the U.S. Army, U.S. Navy, or the Department of Defense.

## Acknowledgments

The Author’s wish to acknowledge Drs. Guofen Gao and Venigalla Rao, The Catholic University of America, Washington DC for providing HIV-1 full-length gp41 protein for the ELISA, and Dr. Francois Villinger New Iberia Research Center, University of Louisianna at Lafayette, New Iberia, LA for providing the antiretroviral therapy.

## Conflict of interest

The authors declare that the research was conducted in the absence of any commercial or financial relationships that could be construed as a potential conflict of interest.

## Publisher’s note

All claims expressed in this article are solely those of the authors and do not necessarily represent those of their affiliated organizations, or those of the publisher, the editors and the reviewers. Any product that may be evaluated in this article, or claim that may be made by its manufacturer, is not guaranteed or endorsed by the publisher.

## Supplementary material

Supplementary Figure 1 Spleens from HIV infected hDRAGA mice that were ART naïve or treated with ART for 42 days and then discontinued ART for 56 days have similar percentages of lymphocytes, FDC, and frequencies of HIV RNA^+^ cells/mm2. Spleen sections were stained with immunofluorescent antibodies to CD20, CD4, CD8, FDC, or *in situ* hybridization for HIV RNA. Percent positive pixel area was determined by visual inspection and quantitative image analysis for each marker and was compared between ART naïve (n=10) and transiently treated (n=5) mice. Bars indicate median values. Statistical analyses were performed using Mann Whitney tests and no differences were found.

Supplementary Figure 2 Gating strategy for flow cytometric analysis of lymphocyte populations from disaggregated spleen tissue of HIV infected and uninfected hDRAGA mice used for subset analyses in **Figure 2**.

Supplementary Figure 3 HIV infected hDRAGA mice demonstrate abnormal lymphocyte organization in lymph nodes. Representative images of mesenteric lymph nodes from **(A-C)** three HIV infected human and **(D-F)** seven HIV infected hDRAGA mice stained for **(A, D)** CD20 (magenta) and DAPI (blue), **(B, E)** CD4 (green) and CD20 (magenta), and **(C, F)** CD8 (green) and CD20 (magenta). Bars equal 200 µm. Graphs depict percentages of **(G)** CD20^+^, **(H)** CD4^+^, and **(I)** CD8^+^ pixel area as determined by visual inspection and quantitative image analysis in CD20^hi^ and CD20^lo^ areas in seven infected hDRAGA mice mesenteric lymph nodes. Statistical analyses were performed using Wilcoxon tests (*p<0.05).

Supplementary Figure 4 Lymphocyte population measurements in spleen from HIV infected (n=11) and uninfected hDRAGA mice (n=7) euthanized more than 250 days post stem cell infusion. Percentages of positive pixels for **(A)** CD4, **(B)** CD8, and **(C)** CD20 were determined by staining spleen sections with immunofluorescent antibodies and quantitative image analysis. Statistical analyses were performed using Mann Whitney tests (*p<0.05, **p<0.01, ns=not significant).

Supplementary Figure 5 hDRAGA mice mesenteric lymph nodes do not form traditional germinal centers and lack canonical FDC distribution. Representative images of HIV infected human (n=3) and hDRAGA mice lymph nodes (n=7) stained for **(A, B)** Ki67 (red) and IgD (green). Bars equal 200 µm. **(C)** HIV infected human inguinal lymph nodes (n=3) were stained for human FDC (yellow) and **(D)** HIV infected hDRAGA mice mesenteric lymph nodes (n=7) were stained for mouse FDC (green) and CD20 (not shown) (n=7). CD20^hi^ areas are demarcated by white lines. Bars equal 100 µm.

Supplementary Figure 6 Spleens from hDRAGA mice contain mouse FDC with aberrant distribution. **(A)** Representative image of normal distribution of mouse FDC in C57/BL6 mouse spleen (n=1). **(B)** Representative image of HIV infected hDRAGA mouse spleen stained with human FDC antibody (n=32). **(C-G)** Representative images of aberrant distribution of mouse FDC in hDRAGA mice spleen (n=32). Spleens were stained with antibodies to CD20 (not shown) and mouse FDC (green). CD20^hi^ areas are demarcated by blue line. No obvious difference in FDC distribution was observed between **(C-E)** HIV infected and **(F, G)** uninfected hDRAGA mice.

Supplementary Figure 7 HIV vRNA^+^ cells and vRNA particles are detected in mesenteric lymph nodes of infected hDRAGA mice. Representative image of *in situ* hybridization for HIV RNA (red) in a **(A)** mesenteric lymph node section from a chronically infected hDRAGA mouse (n=7; shown, mouse 53). Mouse FDC are shown in green. CD20 staining is not shown but CD20^hi^ area is demarcated by green line. vRNA^+^ cells are marked by arrows and representative individual HIV particles are indicated by arrowheads. Bars equal 50 µm and 30 µm on the left and right image, respectively. **(C)** Frequencies of vRNA^+^ cells were quantified in CD20^hi^ and CD20^lo^ areas by visual inspection and quantitative image analysis and **(D)** adjusted for CD4 positive pixel area (n=7). Statistical analyses were performed using a Wilcoxon test (*p ≤ 0.05; ns=not significant).
