# Supplementary figures and images for: HIV-1 infected humanized DRAGA mice develop HIV-specific antibodies despite lack of canonical germinal centers in secondary lymphoid tissues
Source: Front Immunol. 2022 Nov 25;13:1047277. doi: 10.3389/fimmu.2022.1047277 (PMC9732419; doi:10.3389/fimmu.2022.1047277)

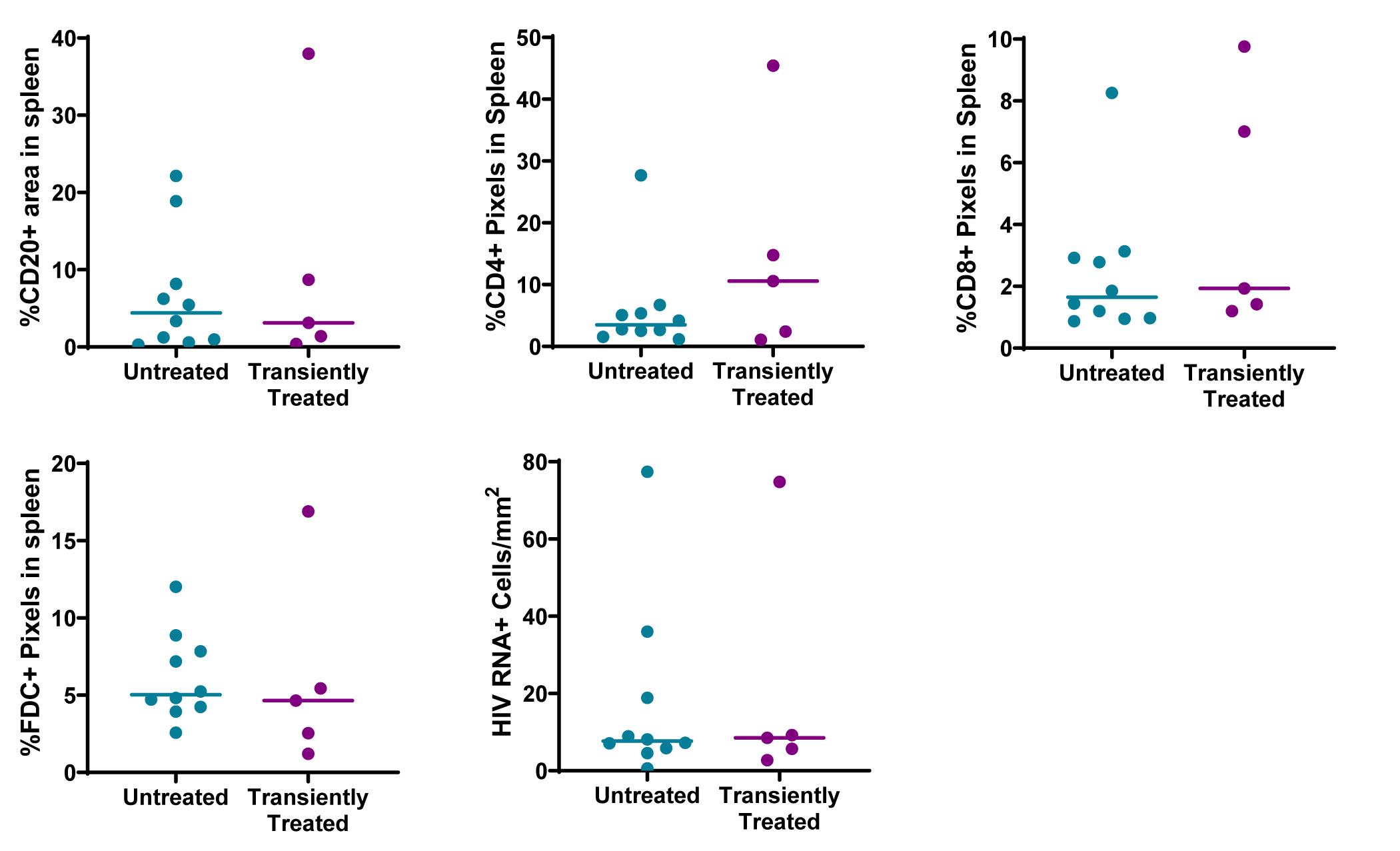

Supplement: Supplementary Figure 1 — Spleens from HIV infected hDRAGA mice that were ART naïve or treated with ART for 42 days and then discontinued ART for 56 days have similar percentages of lymphocytes, FDC, and frequencies of HIV RNA+ cells/mm2. Spleen sections were stained with immunofluorescent antibodies to CD20, CD4, CD8, FDC, or in situ hybridization for HIV RNA. Percent positive pixel area was determined by visual inspection and quantitative image analysis for each marker and was compared between ART naïve (n=10) and transiently treated (n=5) mice. Bars indicate median values. Statistical analyses were performed using Mann Whitney tests and no differences were found. [file Image_1.tif]

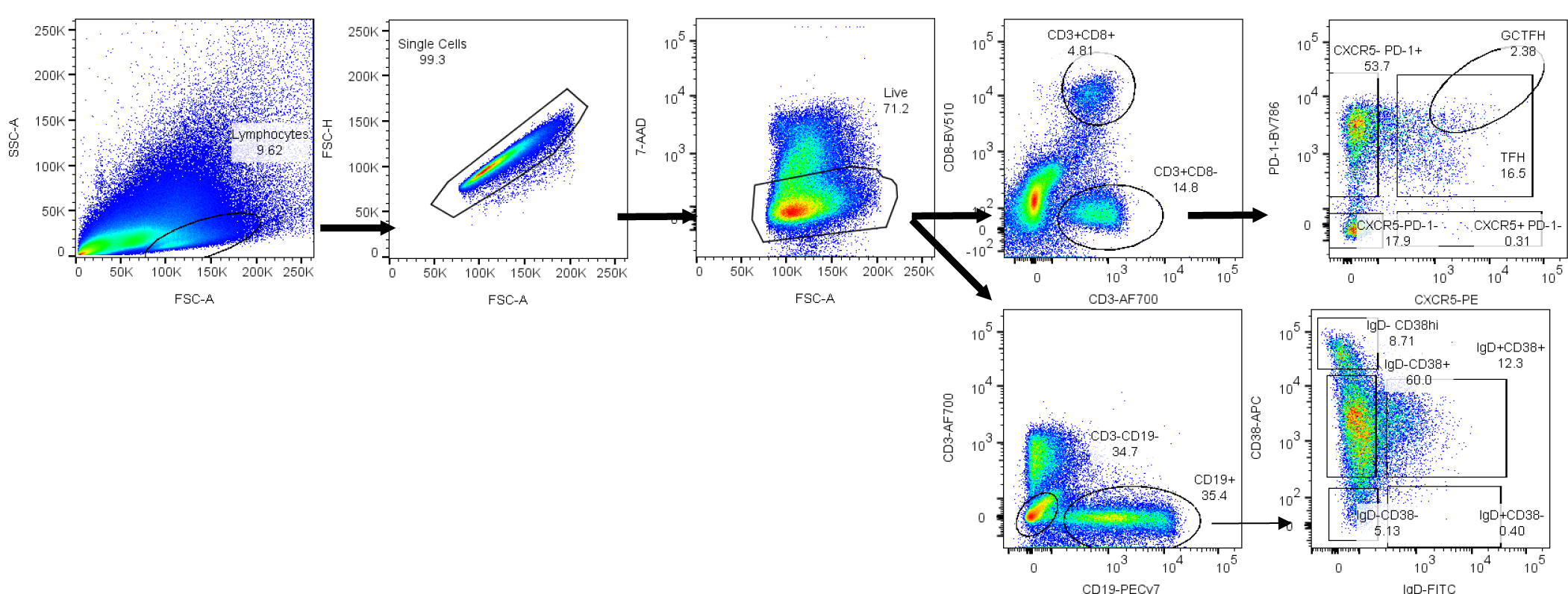

Supplement: Supplementary Figure 2 — Gating strategy for flow cytometric analysis of lymphocyte populations from disaggregated spleen tissue of HIV infected and uninfected hDRAGA mice used for subset analyses in Figure 2 . [file Image_2.tif]

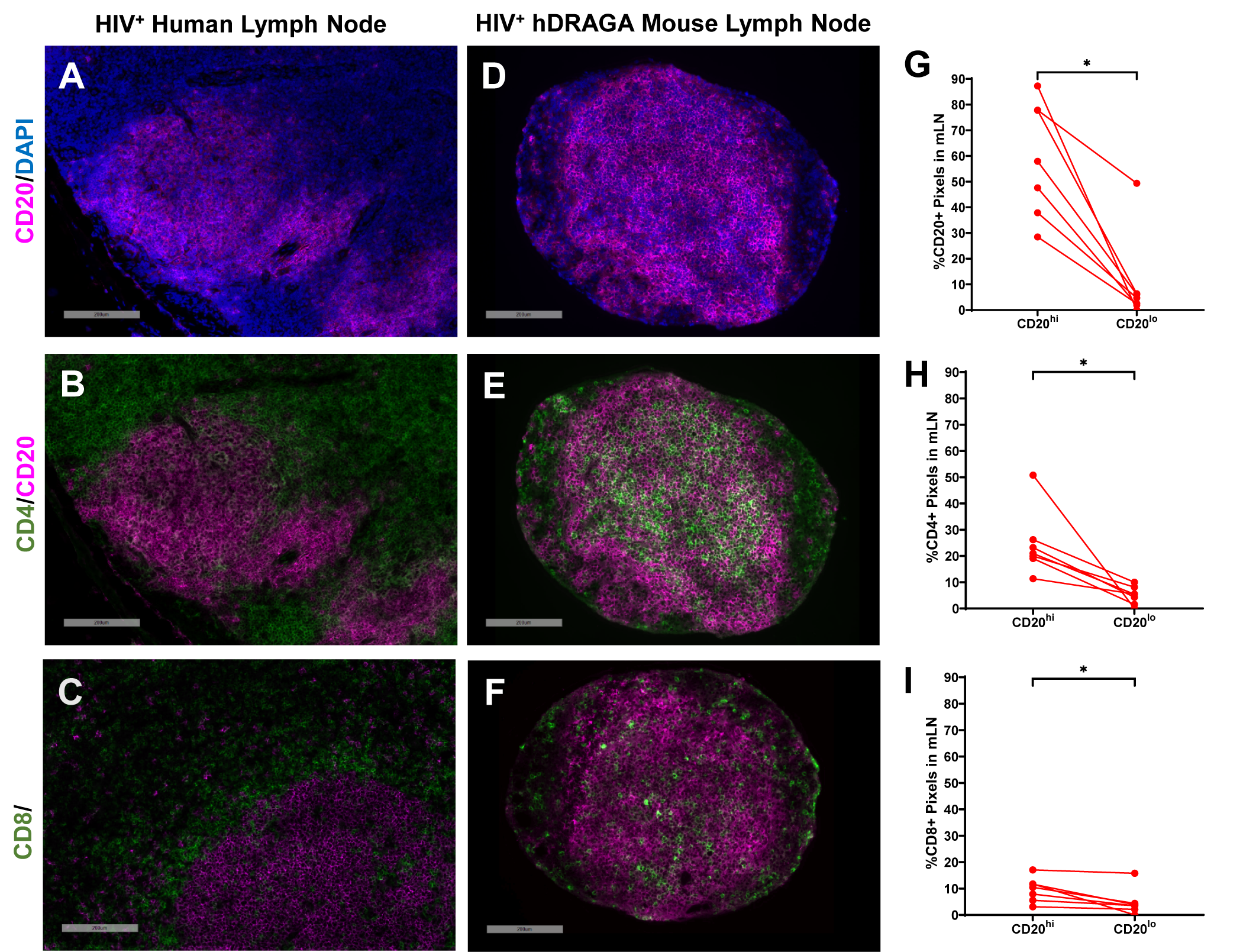

Supplement: Supplementary Figure 3 — HIV infected hDRAGA mice demonstrate abnormal lymphocyte organization in lymph nodes. Representative images of mesenteric lymph nodes from (A-C) three HIV infected human and (D-F) seven HIV infected hDRAGA mice stained for (A, D) CD20 (magenta) and DAPI (blue), (B, E) CD4 (green) and CD20 (magenta), and (C, F) CD8 (green) and CD20 (magenta). Bars equal 200 µm. Graphs depict percentages of (G) CD20+, (H) CD4+, and (I) CD8+ pixel area as determined by visual inspection and quantitative image analysis in CD20hi and CD20lo areas in seven infected hDRAGA mice mesenteric lymph nodes. Statistical analyses were performed using Wilcoxon tests (*p<0.05). [file Image_3.tif]

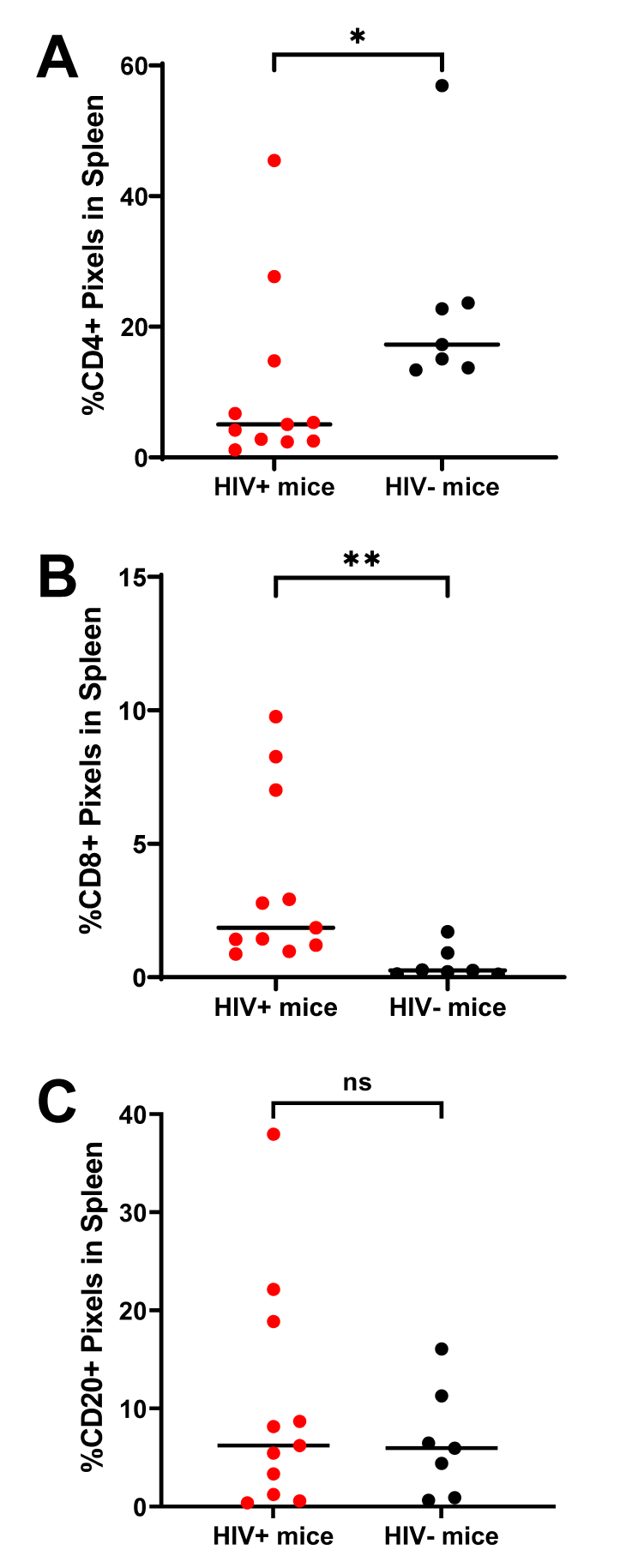

Supplement: Supplementary Figure 4 — Lymphocyte population measurements in spleen from HIV infected (n=11) and uninfected hDRAGA mice (n=7) euthanized more than 250 days post stem cell infusion. Percentages of positive pixels for (A) CD4, (B) CD8, and (C) CD20 were determined by staining spleen sections with immunofluorescent antibodies and quantitative image analysis. Statistical analyses were performed using Mann Whitney tests (*p<0.05, **p<0.01, ns=not significant). [file Image_4.tif]

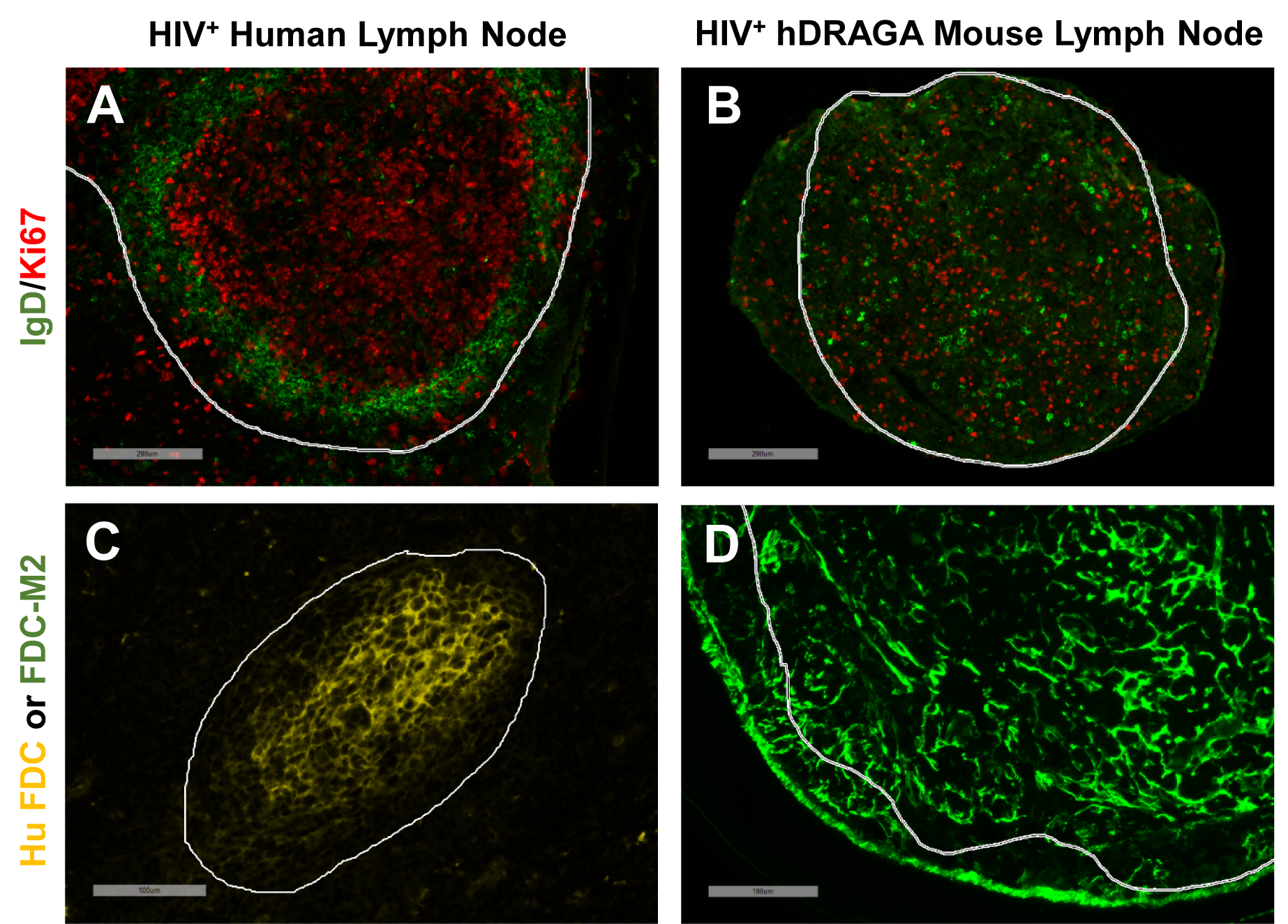

Supplement: Supplementary Figure 5 — hDRAGA mice mesenteric lymph nodes do not form traditional germinal centers and lack canonical FDC distribution. Representative images of HIV infected human (n=3) and hDRAGA mice lymph nodes (n=7) stained for (A, B) Ki67 (red) and IgD (green). Bars equal 200 µm. (C) HIV infected human inguinal lymph nodes (n=3) were stained for human FDC (yellow) and (D) HIV infected hDRAGA mice mesenteric lymph nodes (n=7) were stained for mouse FDC (green) and CD20 (not shown) (n=7). CD20hi areas are demarcated by white lines. Bars equal 100 µm. [file Image_5.tif]

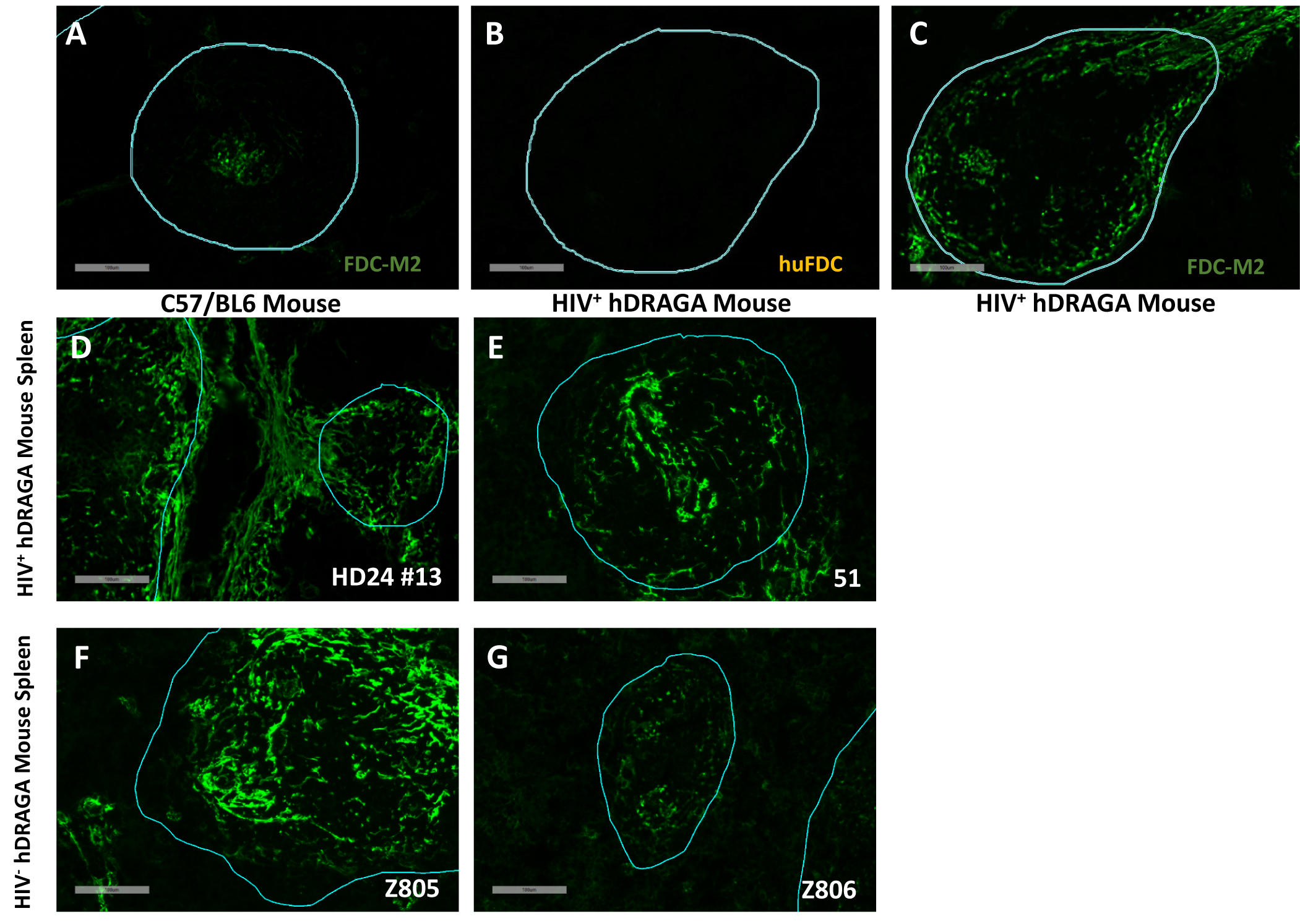

Supplement: Supplementary Figure 6 — Spleens from hDRAGA mice contain mouse FDC with aberrant distribution. (A) Representative image of normal distribution of mouse FDC in C57/BL6 mouse spleen (n=1). (B) Representative image of HIV infected hDRAGA mouse spleen stained with human FDC antibody (n=32). (C-G) Representative images of aberrant distribution of mouse FDC in hDRAGA mice spleen (n=32). Spleens were stained with antibodies to CD20 (not shown) and mouse FDC (green). CD20hi areas are demarcated by blue line. No obvious difference in FDC distribution was observed between (C-E) HIV infected and (F, G) uninfected hDRAGA mice. [file Image_6.tif]

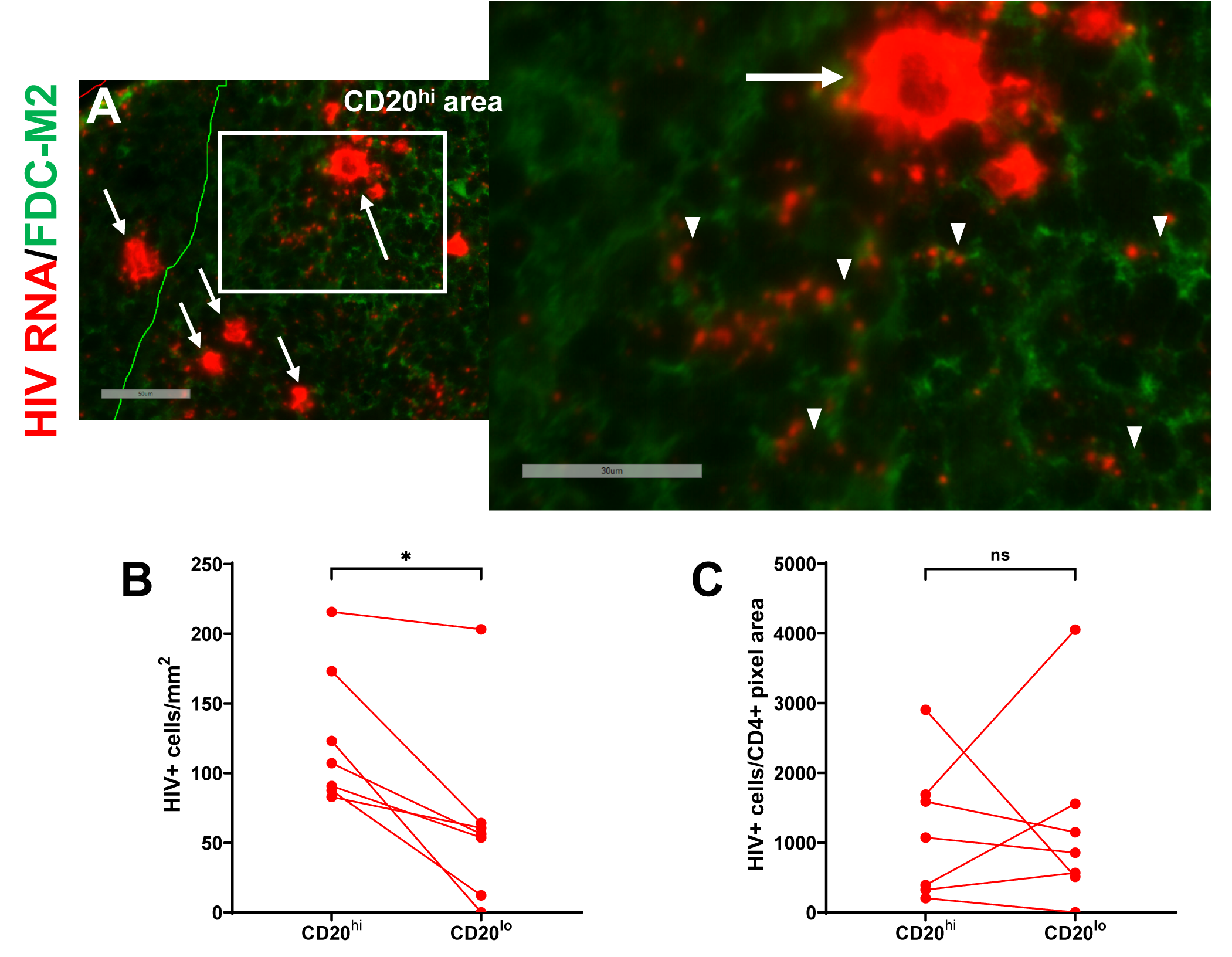

Supplement: Supplementary Figure 7 — HIV vRNA+ cells and vRNA particles are detected in mesenteric lymph nodes of infected hDRAGA mice. Representative image of in situ hybridization for HIV RNA (red) in a (A) mesenteric lymph node section from a chronically infected hDRAGA mouse (n=7; shown, mouse 53). Mouse FDC are shown in green. CD20 staining is not shown but CD20hi area is demarcated by green line. vRNA+ cells are marked by arrows and representative individual HIV particles are indicated by arrowheads. Bars equal 50 µm and 30 µm on the left and right image, respectively. (C) Frequencies of vRNA+ cells were quantified in CD20hi and CD20lo areas by visual inspection and quantitative image analysis and (D) adjusted for CD4 positive pixel area (n=7). Statistical analyses were performed using a Wilcoxon test (*p ≤ 0.05; ns=not significant). [file Image_7.tif]
