# Supplementary material for: HIV-1 infected humanized DRAGA mice develop HIV-specific antibodies despite lack of canonical germinal centers in secondary lymphoid tissues
Source: Front Immunol. 2022 Nov 25;13:1047277. doi: 10.3389/fimmu.2022.1047277 (PMC9732419; doi:10.3389/fimmu.2022.1047277)
Supplement: Supplementary file 8 [file Table_1.pdf]

Supplementary Table 1. Cord blood reconstitution in hDRAGA mice

Mouse	Gender	% hCD19	% hCD3+ CD4+	% hCD3+ CD8+	Donor	Cord blood HLA genotype					
Z32	M	14.7	16.5	1.7	1	A02:01	A11:01	B15:01	B44:02	DR03:01	DR04:01
Z51	M	0.5	17.9	3.6	1	A02:01	A11:01	B15:01	B44:02	DR03:01	DR04:01
Z56	M	1.5	18	2.7	1	A02:01	A11:01	B15:01	B44:02	DR03:01	DR04:01
Z64	M	8.5	9.9	1	1	A02:01	A11:01	B15:01	B44:02	DR03:01	DR04:01
Z81	M	20.1	18.6	5.9	1	A02:01	A11:01	B15:01	B44:02	DR03:01	DR04:01
Z960	M	0.1	38.5	3.8	2	A02:01	A23:01	B15:01	B53:01	DR03:01	DR04:01
Z943	M	1	35.8	2.7	2	A02:01	A23:01	B15:01	B53:01	DR03:01	DR04:01
Z169	F	19.6	14.3	5.7	3	A02:01	A30:02	B07:02	B18:01	DR03:01	DR04:01
Z170	F	25.3	13.3	3.5	3	A02:01	A30:02	B07:02	B18:01	DR03:01	DR04:01
Z805	F	31.6	19.3	0.8	4	A01:01	A02:01	B07:02	B44:02	DR04:01	DR15:01
Z925	F	40.1	22	3.3	4	A01:01	A02:01	B07:02	B44:02	DR04:01	DR15:01
Z806	F	18.6	41.7	2.2	4	A01:01	A02:01	B07:02	B44:02	DR04:01	DR15:01
Z807	F	41.6	23.6	1.7	4	A01:01	A02:01	B07:02	B44:02	DR04:01	DR15:01
Z752	F	37.8	39.7	9.1	5	A02:01	A02:01	B08:01	B44:02	DR04:01	DR03:01
Z739	M	2.7	57.8	5.8	5	A02:01	A02:01	B08:01	B44:02	DR04:01	DR03:01
Z740	M	2.4	56	6.4	5	A02:01	A02:01	B08:01	B44:02	DR04:01	DR03:01
Z758	F	13.2	46.7	4	5	A02:01	A02:01	B08:01	B44:02	DR04:01	DR03:01
Z759	F	32.5	36.2	8.9	5	A02:01	A02:01	B08:01	B44:02	DR04:01	DR03:01
Z742	M	1.6	68.5	5.7	5	A02:01	A02:01	B08:01	B44:02	DR04:01	DR03:01
HD24 #4	F	9.9	34.2	7.4	6	A02:01	A24:02	B08:01	B44:02	DR03:01	DR04:01
HD24 #5	F	3.4	49.8	17.9	6	A02:01	A24:02	B08:01	B44:02	DR03:01	DR04:01
HD24 #6	F	6.3	8.9	5.5	6	A02:01	A24:02	B08:01	B44:02	DR03:01	DR04:01
HD24 #13	F	7.4	12.5	3.5	6	A02:01	A24:02	B08:01	B44:02	DR03:01	DR04:01
HD24 #15	F	6.1	22.7	6.8	7	A02:01	A68:01	B08:01	B44:02	DR03:01	DR04:01
HD24 #25	F	45	9.7	3	8	A02:06	A01:06	B15:06	B49:06	DR07:06	DR04:06
HD24 #26	F	40.8	11.5	3.1	8	A02:06	A01:06	B15:06	B49:06	DR07:06	DR04:06
47	F	56.1	6.4	1	9	A02:01	A11:01	B44:02	B52:01	DR04:01	DR15:02
48	F	40	11.5	2.5	9	A02:01	A11:01	B44:02	B52:01	DR04:01	DR15:02
50	F	31.2	21.6	12.3	10	A02:01	A11:01	B07:05	B40:01	DR0301	DR04:01
53	F	62.2	3.2	0.8	9	A02:01	A11:01	B44:02	B52:01	DR04:01	DR15:02
54	F	39.2	13.7	2.1	10	A02:01	A11:01	B07:05	B40:01	DR0301	DR04:01
59	F	2.7	35.6	4.2	9	A02:01	A11:01	B44:02	B52:01	DR04:01	DR15:02
51	F	48.0	22.9	7.7	10	A02:01	A11:01	B07:05	B40:01	DR0301	DR04:01
52	F	17.8	31	5.9	9	A02:01	A11:01	B44:02	B52:01	DR04:01	DR15:02
55	F	64.1	6.5	2.2	10	A02:01	A11:01	B07:05	B40:01	DR0301	DR04:01
57	F	21.6	11.1	1.7	9	A02:01	A11:01	B44:02	B52:01	DR04:01	DR15:02
58	F	36.8	9	6.1	9	A02:01	A11:01	B44:02	B52:01	DR04:01	DR15:02
